# Professionalization of coaches in performance-oriented sport for athletes with visual impairments: a qualitative study

**DOI:** 10.3389/fspor.2026.1962810

**Published:** 2026-10-06

**Authors:** Ulrich Theobald, Tabea Nauschütz, Jonathan Knapp, Martin Giese

**Affiliations:** 1 Faculty for Special Education, Ludwigsburg University of Education, Ludwigsburg, Germany; 2 Department of Education, Marburg University, Marburg, Germany

**Keywords:** adaptive sport, coaching, elite-level athletes, inclusion, Paralympic sport, professionalization, sports, visual impairment and blindness

## Abstract

**Introduction:**

Coaching athletes with visual impairments (VI) involves distinct pedagogical, sport-specific, and organizational demands. However, research on the professionalization of coaches in Paralympic sport remains scarce. This study explores qualifications, experiences, attitudes, and contextual conditions relevant to coaching in this setting.

**Methods:**

Semi-structured expert interviews were conducted with four experienced coaches working in blind football, goalball, judo, and rowing at a German Paralympic training center. Transcripts were analyzed using structuring qualitative content analysis with an inductive–deductive category-development approach.

**Results:**

Five interrelated dimensions emerged: pathways and motivations for Paralympic coaching, qualifications and competencies, coaching philosophy, institutional conditions, and sport-specific adaptations. Formal qualifications provide an important basis, but disability-specific expertise was primarily acquired through practical experience and self-directed learning. Empathy, flexible communication, individualized motor-learning strategies, and trust-based yet performance-oriented coach athlete relationships were central to practice. Limited resources, informal entry pathways, and insufficient specialist education constrained professionalization.

**Conclusions:**

Coaching in performance-oriented sport for athletes with VI requires more than theory-based licenses. Practice-oriented, disability- and sport-specific education, mentoring, and the systematic transfer of experiential knowledge are needed. Stronger institutional support and collaboration among schools, clubs, and federations may promote sustainable coaching structures and equitable athlete development in Paralympic sport.

## 1 Introduction

The continued growth and professionalization of Paralympic sport have increased the demand for high-quality coaching that can support both athletic performance and long-term athlete development. As Paralympic sport systems expand, coaches play a central role in creating training environments that are performance-oriented, safe, and responsive to athletes' individual needs (1). Understanding how coaches develop the knowledge and competencies required for this work is therefore an important issue for the further evolution of Paralympic sport.

Research on para-sport coach learning suggests that coaches often enter the field through informal or unplanned pathways and develop relevant expertise primarily through practical experience, peer interaction, and mentoring (2, 3). At the same time, formal coach education frequently provides limited disability-specific content and unequal access to specialized learning opportunities (4). These findings indicate that professionalization in para-sport cannot be understood solely in terms of formal qualifications or individual competence, but must also account for biographically contingent entry routes, experiential and relational learning, and the organizational conditions that enable or constrain professional practice (5).

Coaching athletes with VI represents a particularly distinctive professional context (6). Coaches must adapt communication, motor-learning processes, safety arrangements, and instructional strategies to athletes’ sensory conditions and to the requirements of their specific sport (7). Visual demonstrations and conventional spatial cues may be insufficient, requiring alternative approaches to instruction. In addition, sports such as blind football, goalball, judo, and rowing involve different forms of adaptation, support personnel and equipment (8). These demands make it important to examine how general challenges of para-sport coach development are manifested in the specific context of coaching athletes with VI (9).

This study distinguishes between professionalism and professionalization. Professionalism refers to the knowledge, competencies, values, dispositions, and practical judgements through which coaches enact their professional role, including impairment- and sport-specific expertise (10). Professionalization, by contrast, refers to the broader social and institutional process through which coaching becomes increasingly organized, recognized, and supported as a profession, for example through specialized education, formal qualifications, career pathways and institutional recognition (11–13). This distinction enables the study to examine both how coaches develop and enact professionalism in their everyday practice and how organizational conditions enable or constrain the professionalization of para-sport coaching.

Although previous research has identified informal learning pathways, gaps in disability-specific coach education, and the importance of experiential learning in para-sport coaching, limited evidence is available on how these processes are shaped by the specific demands of coaching in performance-oriented sport for athletes with VI. In particular, little is known about how coaches across different VI sports develop and apply relevant expertise within the German Paralympic system. Accordingly, this study addresses the following research question: How do qualifications, professional experiences, orientations, and organizational conditions shape the professionalization of coaches working in performance-oriented sport for athletes with VI in Germany?

## 2 Methodology

As was mentioned before, research on the professionalization of coaches working with elite athletes with VI remains limited. We therefore adopted an exploratory qualitative design to examine multiple dimensions of coaching professionalism in this domain. In order to gather insight in their day-to-day professional practice, we conducted interviews with experienced coaches in order to access their specialist knowledge (14). We contacted all seven coaches employed at one of the largest Paralympic training centers for athletes with VI in Germany by e-mail and invited them to take part in the study. Of the invited population (*n* = 7), four coaches agreed to participate, responsible for blind football, goalball, judo, and rowing. This equals to a proportional non-response of 43%. Participants reported 5–19 years of coaching experience (*M* = 13.25 years), indicating substantial domain expertise.

Although the coaches were employed in a Paralympic training-center context and contributed to performance-oriented sport development, their responsibilities extended across the athlete pathway, including children and younger athletes, developing competitors, and athletes pursuing national or Paralympic-level performance. All participants hold a professional pedagogical qualification or a higher-education teaching degree.

### 2.1 Data collection and analysis

Data collection and analysis followed a systematic, theory-based qualitative procedure designed to capture the complexity of coaches' professional knowledge and practice (15). We conducted semi-structured systematizing expert interviews (16), a method particularly suited to accessing specialized, experience-based knowledge while maintaining sufficient structure to enable cross-case comparison (14). Because this format relies on a clear balance between openness and guidance (17), the interview guide was developed with particular care: It was structured around theoretically derived categories grounded in the literature on professionalism in coaching, covering core dimensions such as qualifications, role conceptions, everyday practices, and contextual conditions (11, 12). Prior to data collection, the guide was discussed collegially among all authors to ensure conceptual coherence, topical relevance, and alignment with the study's exploratory aims (18). A pre-test was conducted with a PE teacher with experience in coaching Paralympic sports who was employed at the main author's department during the study's time-frame. For this purpose, a cognitive interview was used to assess the consistency and comprehensibility of the interview questions after which only wording and sequence of the interview questions were slightly refined (19).

Interviews were conducted in German language between February and May 2026 in the coaches’ offices by the second and third author. Both are currently research assistants in the field of sports pedagogy, have a background in adaptive sport and are experienced in qualitative interviewing. Each of them conducted two of the interviews individually and in person. The interviewers did not have any prior relationship to the participants. The interview procedure was discussed in advance as a team to align on objectives, questioning strategies, and strategies for building rapport with participants (20). We adopted an open, flexible interviewing approach that permitted free-flowing conversation within the predefined thematic framework, allowing interviewees to introduce unanticipated but relevant topics while ensuring coverage of all key dimensions (18). Throughout the data collection phase, a fieldwork diary with memos was maintained to document contextual observations, methodological reflections, and emerging analytic ideas, thereby supporting transparency and reflexivity in the research process (20). Those were not formally included in the review process. Interviews lasted between 45 and 90 min.

Interview recordings were transcribed in German language using artificial intelligence-supported transcription software (Whisper, locally installed) and subsequently checked and corrected line-by-line by the first author and a research assistant to ensure accuracy and fidelity to the original speech. The quotations used in this manuscript were translated by the main author and checked for consistency by the other authors. An initial category system was developed based on the aforementioned dimensions of professionalism and then iteratively refined through close engagement with the transcripts. During the initial coding cycle, the deductive framework was used to organize any material related to the established dimensions of coaching professionalization. At the same time, text segments that introduced aspects not sufficiently represented in this framework were marked and assigned provisional open codes. These codes were compared across the four interviews and clustered according to recurring similarities in meaning. Through repeated review of the coded passages, the provisional codes were condensed, differentiated, or merged into inductively developed subcategories. The resulting subcategories were subsequently positioned within the broader theoretical categories, thereby allowing the final analytical structure to retain its theory-informed orientation while representing context-specific features of coaching in performance-oriented sport for athletes with VI (21). This system was discussed in detail with the second and third author as well as another research assistant to refine category definitions, resolve ambiguities, and ensure interpretive consistency across the team. The resulting category system included both theory-derived (deductive) and data-driven (inductive) categories, which are presented in the results section alongside a visual overview of the final category system (20).

The study employed a collaborative coding approach in which multiple researchers discussed category definitions, considered alternative interpretations, and negotiated coding decisions to promote interpretive consistency rather than to calculate statistical interrater agreement (22). This procedure is consistent with recommendations for exploratory qualitative designs with small datasets (23), prioritizing theoretical sensitivity, depth of interpretation and contextual understanding over statistical reliability measures (24). All transcripts were coded by the first author and a research assistant, with coding decisions negotiated through discussion.

## 3 Results

The interview data were analyzed using structuring qualitative content analysis (20), resulting in five main categories that reflect the experiences, perspectives, and practices of the coaches who were interviewed in this study. The first category, pathways and motivation for Paralympic coaching (I), addresses their personal routes into this field and their underlying motivations for engagement. The second category, qualification and competencies (II), examines the educational background, knowledge, and skills regarded as essential for coaching athletes with VI.

Building on this, coaching philosophy and pedagogical approach (III) captures coaches' guiding principles with respect to athlete involvement, personal responsibility, and inclusion. Competencies were coded when participants referred to knowledge, skills, capacities, or practical abilities required to coach athletes with VI (e.g., empathy, flexible communication, disability-specific knowledge, or the ability to adapt instruction). Coaching philosophy and pedagogical approach were coded when participants articulated normative principles, values, or guiding assumptions concerning how coaching should be enacted (e.g., athlete autonomy, performance orientation, appropriate challenge, trust, inclusion, or the balance between authority and facilitation).

The fourth category, institutional and organizational framework (IV), considers the structural conditions that shape and sustain Paralympic sport development. Finally, sport-specific adaptations for visually impaired athletes (V) highlights the training related requirements and modifications that individual sports entail to accommodate athletes with VI. Sport-specific adaptations were coded separately when participants described concrete impairment- or sport-related modifications to communication, safety arrangements, equipment, rules, or motor-learning processes.

Collectively, these five categories provide a comprehensive account of the multifaceted role of coaches in Paralympic sports for visually impaired athletes (Table 1).

**Table 1:** Category system with subcategories, operational definitions, and anchor examples.

Main category	Subcategory	Operational definition	Anchor example
Pathways and motivation for Paralympic coaching	Initial contact and entry pathways	Biographical, educational, professional, or network-based circumstances through which coaches first encountered disability sport, visual impairment, or Paralympic coaching.	“*I completed an internship there - during my first semester and later, I think, over a summer term. Or perhaps for an entire semester, I supported a class together with the PE teacher there. We simply taught PE classes and carried out projects at the time. That was how I got to know the school and came into contact with blindness*” (I02).
	Intrinsic and relational motivation	Enjoyment derived from athletes’ enthusiasm, coach–athlete relationships, participation, community, and the opportunity to make sport accessible.	“*This connection with visually impaired and blind children, giving them the opportunity to do this sport and, through this sport, also to build a sense of community. I think that really excites me*” (I03).
	Commitment to sport development and performance pathways	Motivation to establish, professionalize, or sustain clubs, leagues, competitions, talent pathways, or Paralympic opportunities.	“*If a few good ones develop in such a way that they eventually make it to the Paralympics, that's just awesome. But that can only grow out of a strong, broad grassroots base*” (I02).
Qualifications and competencies	Formal qualifications and disability-specific education	General pedagogical, sport-science, or coaching qualifications, as well as the availability, perceived adequacy, and practical relevance of disability- and sport-specific education.	“*Well, of course, I have a coaching license*” (I03).
	Self-directed and experiential learning	Knowledge acquired through practical engagement, trial and error, reflection, observation, and learning from experienced practitioners rather than through formal education alone.	“*I do think that this is a qualification in its own right. But it is all learning by doing and self-taught*” (I04).
	Relational and personal competencies	Capacities to understand athletes’ individual situations, establish trust, respond empathetically, and exercise context-sensitive interpersonal judgement.	“*You have to meet the children where they are. Particularly those who are Para-athletes are incredibly diverse*” (I03).
	Adaptive instructional competencies	Capacities to modify communication, feedback, tasks, and instructional procedures in response to individual sensory, cognitive, and sporting conditions.	“*If we leave aside the didactical aspect for a moment, the way you work is very different when you are explaining something to someone who is blind. That is where it begins: I cannot simply say, ‘Look, I’m going to show you something now’*” (I03).
Coaching philosophy and pedagogical approach	Athlete-centered development and autonomy	Normative orientation towards involving athletes in decision-making, fostering personal responsibility, and supporting them as active participants in their own development.	“*I try to take a democratic approach. I try to involve them throughout the whole process and encourage them to take responsibility*” (I01).
	Trust, authority, and coach-athlete relationship	Principles guiding the balance between professional authority, relational closeness, mutual respect, and trust in coaching relationships.	“*Nothing bad has ever happened, and that creates trust at a very intense and very profound level*” (I03).
	Performance orientation and appropriate challenge	Commitment to demanding, performance-oriented coaching that avoids both lowered expectations and overprotection while taking individual conditions into account.	“*Here, nobody is checked for who has no chance of making it to the highest division and then kicked out. I do think that there are certainly very good examples in Olympic youth centers. But this absolute focus on performance is, in our context, defined somewhat differently, I believe*” (I04).
	Inclusion and non-paternalistic practice	Orientation towards treating athletes with VI as athletes and sporting agents, rather than primarily through an impairment-focused or protective lens.	“*The impairment is also part of this sport. It is not that it shouldn't be mentioned, but nobody wants to hear about the person's great suffering in their life and all that stuff*” (I02).
	Institutional cooperation and network structures	Cooperation among schools, clubs, federations, disability-sport organizations, support associations, and training centers in delivering coaching and athlete pathways.	“*That people do not undermine each other, that they do not economically threaten one another's very right to exist, but instead find a way to create cooperation*” (I01).
Institutional and organizational framework	Development of sport and talent structures	Establishment or consolidation of clubs, leagues, competitions, youth centers, support organizations, and talent pathways.	“*They established a youth performance center several decades ago*” (I02).
	Resources, infrastructure, and staffing	Financial, spatial, material, personnel, and accessibility-related conditions that enable or constrain coaching and sport development.	“*…because they had also recognized the need for it. So we established a support association*” (I02).
	Multiple professional roles	The combination of teaching, coaching, management, administration, and organizational-development tasks undertaken by teacher-coaches.	“*It developed through overlaps with the club's judo section which I run. As part of that, I am also responsible for talent development at the school. That more or less describes all of my responsibilities. I take care of all the organizational matters - essentially everything - because there is no one else besides me*” (I03).
Sport-specific adaptations for athletes with VI	Safety, orientation, and support personnel	Adaptations intended to support safe participation and spatial orientation, including guides, scouts, verbal commands, environmental adjustments, and protective arrangements.	“*I can't speak for the others. We always need guides; it simply doesn't work without them. Safety is a huge aspect, and of course that slows things down*” (I01).
	Adapted rules, equipment, and training arrangements	Sport-specific adjustments to equipment, rules, starting positions, settings, and the organization of training or competition.	“*In my view, judo is the only sport in which para-athletes can train and participate alongside non-disabled athletes, because all other sports are heavily regulated*” (I03).
	Non-visual communication and feedback	Use of auditory, tactile, kinesthetic, and explicit verbal strategies where visual demonstrations or conventional spatial cues are insufficient.	“*You are not standing half a meter or a meter apart from one another; instead, you take hold of each other and establish physical contact, which removes all barriers*” (I03).
	Motor learning and perceptual conditions	Consideration of altered perceptual conditions, movement acquisition, orientation, and sport-specific motor learning among athletes with VI.	“*Because these are overarching ideas – essentially overarching concepts such as how movement acquisition and motor learning work in the context of blindness. That is certainly different from how it works for sighted people*” (I04).

### 3.1 Pathways and motivation for Paralympic coaching

This category encompasses coaches' personal pathways into Paralympic sports and their motivations for engagement. It includes aspects such as the first contact with the topic of blindness or VI, the initial fascination with sport for people with disabilities, and the intrinsic or extrinsic factors that led to taking on a coaching role, such as the athletes' enthusiasm or the opportunity to make sport accessible.

Coaches' initial contact with disability sport or blindness as well as VI in sport typically occured through incidental circumstances or professional and academic trajectories rather than deliberate career planning. Entry points ranged from teacher-training programs and additional qualifications to pre-existing roles in adjacent sports that connected these coaches with specialized institutions through personal or professional networks. In several cases, initial engagement began as a limited, one-off activity that only evolved into sustained involvement once a leadership or management role was assumed. A shared affective element across accounts is an initial sense of fascination or surprise at witnessing the athletic capabilities of individuals with VI, which appears to have fostered their longer-term commitment despite early logistical or pedagogical difficulties.

Motivation for coaching engagement: Motivation is predominantly intrinsic and draws on several converging sources. The most prominent is a commitment to enabling participation and accessibility—coaches frame their role as opening sporting opportunities that would otherwise remain inaccessible to individuals with impairments, positioning themselves as facilitators of inclusion rather than mere instructors: “*this connection with visually impaired and blind children, giving them the opportunity to do this sport and, through this sport, also to build a sense of community. I think that really excites me*” (I03). A second source is relational and affective: The satisfaction coaches derive from athletes' enthusiasm and from the coaching relationship itself functions as a reward that sustains long-term engagement. A third, more structural motivation concerns the professionalization and institutional development of the sport. Coaches who entered fields lacking established competitive or organizational structures describe substantial personal investment of time and creative problem-solving as a response to this gap, framing their contribution partly in terms of building durable structures and professional networks rather than solely coaching individual athletes.

Unique aspects and challenges: Beyond these shared patterns, individual trajectories reveal sport-specific institutional histories, including moments of structural change (e.g., shifts in organizational leadership) that catalyzed further professionalization, as well as sport-specific adaptations required for safe and effective practice. Aspirations toward elite-level competition, including Paralympic participation, also emerge as a motivating horizon that shapes coaches’ long-term orientation, often coupled with an awareness of resource constraints that necessitate improvisation and creative solutions: “*If a few good ones develop in such a way that they eventually make it to the Paralympics, that's just awesome. But that can only grow out of a strong, broad grassroots base. It just can't work otherwise. Honestly, why have I been doing this for 20 years? Because I enjoy it*” (I02).

### 3.2 Qualification and competencies

Topics identified in this main category encompass the formal education, autodidactic knowledge, and personal as well as professional skills considered essential by the participants. These include aspects such as coaching licenses, sports science degrees, and additional qualifications in sports for the visually impaired, as well as practical experience, insight into human nature, empathy, flexibility, and the ability to individually support and challenge athletes.

Formal qualifications and self-directed learning: The interview partners agree, that coaches in Paralympic sport typically hold a general sport-related formal qualification (e.g., university degrees, coaching licenses), yet report that disability-specific certifications are rare and often perceived as overly theoretical or insufficiently practice-oriented. As a result, they think that core expertise for coaching athletes with disabilities is largely developed through self-directed, experiential learning. This “learning by doing” is described as central to understanding athletes' lived realities and to building practically relevant coaching competence beyond what formal training currently provides. Despite the centrality of autodidactic learning, the coaches we interviewed express a clear need for specialized initial and continuing education programs. Furthermore, they call for more practice-oriented, disability-specific programs and licenses, ideally designed and delivered by experienced Paralympic coaches. They also express interest in formal structures that would allow their accumulated experiential knowledge to be systematically shared with new generations of coaches.

Across the interviews, essential competencies are primarily personal and relational. Empathy, social competence, and nuanced knowledge of people are repeatedly emphasized, together with a deeper understanding of blindness, VI and related theories of perception: “*Especially with younger athletes, the empathic component is incredibly important, because you have to meet the children where they are. Particularly those who are Para-athletes are incredibly diverse*” (I03). These competencies are seen as foundational for establishing trust, reading individual needs, and responding adequately to the specific experiential worlds of Paralympic athletes.

Flexibility and individualization are described as crucial didactic and methodological requirements. Coaches underline the need to “pick up” athletes at very different starting points and to adapt communication and instruction to diverse sensory and cognitive conditions. Conventional instructional routines (such as visually anchored cues) must be reworked, and rigid dogmas replaced by individually tailored pathways, in order to ensure both safety and effective skill acquisition.

### 3.3 Coaching philosophy and pedagogical approach

This category encompasses philosophies and methods of the interview partners in working with their athletes, particularly with regard to development, personal responsibility, and inclusion. It includes aspects such as involving athletes in decision-making processes, challenging rather than sheltering them, emphasizing sport and performance, imparting fundamental motor skills, and building a trusting teacher-student relationship.

Coaching philosophy and athlete development: Across participants' accounts, coaches share a core philosophy of meeting athletes where they are and fostering their individual development so they can realize their full potential. This entails a deliberate refusal to overprotect athletes: instead, coaches emphasize trusting athletes' capabilities, exposing them to challenge and, where necessary, to critical feedback. Normative demands are seen as appropriate and as a counterpoint to more overprotective pedagogical styles often encountered in disability contexts. Coaches articulate a preference for fostering athletes' autonomous engagement and potential rather than encouraging compliance with instructions, suggesting an underlying pedagogical model oriented toward empowerment and mutual respect between coach and athlete.

Inclusion and dealing with disability constitute focal points of coaching practice as described in the interviews. Coaches aim to integrate athletes with VI or other disabilities into mixed training groups under equal conditions, with comparable tasks and responsibilities. While impairment is acknowledged as part of the sporting context, it is not positioned as the dominant lens; rather, athletes with disabilities are treated in principle like athletes in the Olympic sector. Coaches frame their role as opening up sporting opportunities that would otherwise be inaccessible.

The role of the coach is conceptualized as a blend of authority, trust, and facilitation. Building a strong trust basis is described as essential, particularly in disciplines such as martial arts where physical risk is inherent. Coaching is understood as combining clear presence and authority with mutual respect and relational closeness. Some coaches adopt a markedly democratic approach, involving athletes in decision-making processes, transferring responsibility, and defining themselves less as superiors than as enablers. Others, especially in school-based settings, retain a more traditional teacher–student structure with younger athletes, while moving towards more collaborative relations with older ones.

Performance orientation: A consistent stance across accounts in the data favors a performance-oriented, developmental approach that treats athletes with disabilities as capable competitors. All coaches reject overly protective or “*cushy pedagogies*” (I02) and criticize lowered expectations based solely on impairment, insisting Paralympic athletes be treated as full athletes with demanding, performance-centered training, while still acknowledging individual constraints rather than over-thematizing them. This shared orientation nonetheless translates into differing approaches to system development. Some coaches in our study have driven strong professionalization efforts: founding clubs, leagues, and support structures and envisioning self-sustaining systems built on continuous competitive pressure, mirroring high-performance sport logics. Others affirm performance's importance while rejecting exclusionary selection practices common in Olympic youth development, favoring athlete-retention over sorting “*Here, nobody is checked for who has no chance of making it to the highest division and then kicked out. I do think that there are certainly very good examples in Olympic youth centers. But this absolute focus on performance is, in our context, defined somewhat differently, I believe*” (I04). A related critique targets mechanistic, grid-based performance systems that neglect intrinsic motivation and holistic development, suggesting performance and athlete-centered growth need not conflict.

### 3.4 Institutional and organizational framework

The institutional framework conditions, collaboration with other organizations, and the development of sport structures in the Paralympic domain are all subthemes of this main category: It includes the role of schools, sports clubs, state and national federations, the establishment of support associations or leagues, as well as the creation of youth development and performance centers that contribute to the promotion of the sport.

The interviews depict organizational structures and collaborations in German Paralympic sport as the product of dynamic development driven by a small number of highly engaged actors. The coaches' proprietary training center emerges as a central hub, functioning as an over-regional support center for blind and visually impaired people and as a focal point for talent concentration and the development of Paralympic sports structures. Through infrastructure provision and the release of teacher-coaches, it serves as a key institutional partner in establishing youth and performance centers.

Sports structures have been progressively built and professionalized via several mechanisms: the creation of national leagues and competitions (e.g., in goalball and blind football), the founding of support associations and umbrella organizations to pool administrative and financial responsibilities, and the establishment of Paralympic youth centers and elite-school arrangements that anchor talent development. Collaboration with national federations and disability sport organizations is crucial for institutional embedding and funding, linking school-based initiatives with the broader sport system. Despite notable progress, persistent challenges remain: high costs and limited financial recognition, infrastructural and accessibility issues, constrained staffing and support ratios, and friction in coordination with athletes' wider environment (caregivers, parents, schools). At the same time, synergies between institutions, voluntary engagement within the community are described as core resources for ongoing professionalization: “*That people do not undermine each other, that they do not economically threaten one another's very right to exist, but instead find a way to create cooperation*” (I01).

A recurring pattern is the central role of teacher-coaches who hold multiple concurrent professional roles, often combining teaching, coaching, and broader managerial responsibilities. These individuals are key drivers of structural innovation and sporting success but shoulder considerable administrative and organizational workloads.

### 3.5 Sport-specific adaptations for visually impaired athletes

This category encompasses the specific requirements and adaptations that the interview partners deem as necessary for people with VI. It includes aspects such as the integration of visually impaired and non-visually impaired athletes, the importance of safety (e.g., through guides/scouts), the adaptation of rules or starting points, the need for special equipment, and the effects of VI on motor learning and perception in sport.

Safety and specific adaptations are described as a transversal concern, shaping both infrastructure and training design. Certain sports require additional personnel (e.g., scouts) and modified equipment, while others rely on adjusted starting positions or protective measures in the environment. In ball sports and rowing for example, structured preparatory exercises and explicit verbal commands are used to support orientation and reduce collision risk: “*I can only speak about athletes with VI. I can't speak for the others.* [In rowing] *we always need guides; it simply doesn't work without them. Safety is a huge aspect, and of course that slows things down*” (I01).

Motor learning and didactics require tailored approaches due to potentially altered motor development and different perceptual conditions. Visual demonstration is often insufficient, necessitating alternative instructional strategies and exercises that simultaneously build technical skills as well as orientation. “*Because these are overarching ideas – essentially overarching concepts such as how movement acquisition and motor learning work in the context of blindness. That is certainly different from how it works for sighted people*” (I04).

Finally, psychological aspects form a distinct subtheme within the interviews. Some interview partners point to the particular nature of athletes' biographies, while others stress their desire to be recognized primarily for sporting performance rather than their impairment or potentially associated pity. Together, these elements illustrate the multifaceted, sport- and context-specific demands of coaching and organizing Paralympic sport for visually impaired athletes, which require nuanced handling within a developmental, performance-oriented coaching philosophy.

## 4 Discussion: integrating the main categories

Taken together, the five categories identified in this study illustrate that coaching in Paralympic sports for visually impaired athletes is shaped by a complex interplay of personal, structural, and sport-specific factors, none of which can be fully understood in isolation. Rather than operating as discrete determinants of coaching practice, these dimensions appear to be mutually reinforcing (25). Deficits in one domain, for instance the absence of formal qualification pathways, are frequently compensated for through informal mechanisms in another, such as sustained practical experience, mentoring, or strong institutional embedding (26). This pattern resonates with broader debates in the coaching science literature on the limits of formalized, competency-based education models when applied to highly specialized or under-resourced sporting contexts (27, 28).

Recent research has identified informal entry pathways, uneven access to formal coach education, and the central role of experiential and relational learning as recurring features of para-sport coach development (4, 5, 9). Accordingly, the present findings do not suggest that these challenges are unique to coaching athletes with VI. Rather, the contribution of this study lies in specifying how general processes of coach learning and professionalization are shaped by the sensory and didactic demands of VI.

### 4.1 Biographically contingent pathways to professionalization

A first cross-cutting finding is that professionalization in this field is often biographically contingent rather than organized through a linear or clearly formalized career pathway. Participants' engagement in Paralympic coaching frequently originated in chance encounters, personal fascination, or incidental exposure to blindness or VI through teacher education, previous professional roles, or personal networks. Entry into the field was therefore less commonly described as the outcome of deliberate career planning than as a gradual process in which an initially limited engagement developed into sustained coaching responsibility.

This pattern diverges from more institutionalized coaching trajectories in mainstream elite sport, where certification sequences and talent-pathway programs may precede professional practice (29, 30). In the present study, professional development was instead characterized by the progressive accumulation of practical knowledge within specific sporting and institutional contexts. Formal qualifications and general coaching licenses provided an important basis, but participants consistently described disability-specific expertise as being acquired through self-directed learning, repeated practical engagement, and reflection on the lived realities of athletes with VI. The findings thus support the argument that para-sport coaching knowledge cannot be reduced to formal certification, particularly where specialist education remains limited or insufficiently practice-oriented.

This finding is consistent with Fairhurst et al. (3), who identified limited opportunities to acquire disability-specific knowledge through formal education and emphasized the importance of experiential learning and mentoring in Paralympic coaches' development. Alexander and Bloom (2) similarly showed that mentoring can provide para-sport coaches with access to disability-specific knowledge, professional exchange, and a sense of community. Although mentoring was not uniformly available as a formal structure in the present study, participants' emphasis on learning from experienced practitioners and their interest in passing on accumulated knowledge suggests that comparable functions were fulfilled informally and unevenly. The current findings also align with Townsend et al. (9) and Simoni et al. (4), who argue that formal, informal, and experiential forms of coach learning need to be considered together rather than treated as competing alternatives.

The reliance on informal entry points and individually accumulated knowledge may constrain both the size and diversity of the available coaching workforce. It makes recruitment dependent on serendipitous circumstances and risks leaving valuable experiential knowledge embedded in the biographies of individual coaches rather than making it accessible as a shared professional resource. More visible and structured entry routes, for example through teacher-training programs, sport science curricula, disability-sport organizations, and partnerships with schools for the visually impaired, may therefore be important for making entry into the field less dependent on chance.

### 4.2 Relational and impairment-specific professionalism

A second argument concerns the relational and impairment-specific character of professionalism in coaching athletes with VI. Across the interviews, social competence, flexibility, and nuanced knowledge of individual athletes were not presented as supplementary “soft skills,” but as core conditions for safe, performance-oriented, and developmentally appropriate coaching. Participants described the need to recognize highly diverse biographical, sensory and cognitive starting points and to adapt their communication and expectations accordingly.

This relational work was closely connected to a coaching philosophy that combined trust, athlete involvement, and high-performance expectations. Coaches rejected an overprotective approach that would define athletes primarily through impairment or lower expectations on that basis. Instead, they sought to recognize athletes with VI as capable sporting agents, to involve them in decision-making, and to expose them to appropriate challenge and critical feedback. Trust therefore appears not merely as a desirable feature of the coach–athlete relationship, but as a practical precondition for combining individual sensitivity with demanding training and performance standards. This interpretation is compatible with research emphasizing the centrality of relational quality, communication, and individualized support for training quality in elite para-sport settings (31).

However, relational professionalism in this context cannot be separated from impairment-specific and sport-specific pedagogical expertise. Conventional visually anchored demonstrations and spatial cues may be insufficient for athletes with VI, requiring coaches to use alternative forms of instruction and feedback. Safety-related arrangements, such as the use of guides or scouts, modified equipment, adapted starting positions, and explicit verbal orientation cues, are therefore not merely technical additions to generic coaching. They fundamentally shape how training is organized, how movement is learned, and how coach-athlete communication is enacted in practice.

The findings consequently suggest that expertise in coaching athletes with VI involves the continuous translation of general coaching knowledge into individualized, sensory-responsive, and sport-specific practice. In this sense, adapted communication and motor learning should be understood as integral dimensions of professional judgement rather than as peripheral disability-specific adjustments. This is in line with work that highlights the importance of context-sensitive, athlete-centered training environments and adaptive coaching practices in para-sport (31, 32). The present study adds that, for coaching athletes with VI, such adaptation involves not only technical knowledge but also the relational capacity to interpret athletes' individual needs without reducing them to their impairment.

### 4.3 Professionalization as an organizational process

A third overarching finding is that professionalization should not be understood solely as an individual process of acquiring qualifications and competencies. In the present study, coaches' capacity to develop and apply specialist expertise depended substantially on organizational structures, institutional collaboration, and recruitment opportunities. Schools for students with VI, sports clubs, national and regional federations, youth development structures, and Paralympic training centers were described as interdependent actors in the development of sustainable coaching and athlete pathways.

The Paralympic training center in this study functioned as more than a physical site of practice. It concentrated athletes, infrastructure, and professional networks and thereby created opportunities for knowledge exchange, talent development, and collaboration across educational and sport organizations (33). At the same time, persistent limitations in funding, staffing, accessibility, and coordination with athletes' wider environments constrained the development of these structures. The findings thus indicate that individual dedication and self-taught expertise cannot fully compensate for insufficient organizational support, particularly in a small and specialized para-sport workforce.

This organizational perspective is especially important given the multiple roles assumed by teacher-coaches in the present sample. Their work frequently combined teaching, coaching, administration, and structural development. These roles were central to sporting development and institutional innovation, but they also created substantial additional workloads that are not always formally recognized. Professionalization therefore involves not only strengthening individual coaches' competencies, but also creating employment, funding, and coordination arrangements that make such work sustainable.

This interpretation corresponds with Huntley et al. (5), who conceptualize the European para-sport coaching workforce as a quasi-professional space shaped by structural conditions as well as individual capabilities. It also supports the review by Simoni et al. (4), which emphasizes the need to combine formal, informal, and experiential learning opportunities within supportive organizational settings. In the present context, schools, clubs, federations, and Paralympic training centers need not simply provide separate services; they need to cooperate in creating visible recruitment pathways through which practical knowledge can circulate beyond individual coaches and local sites.

### 4.4 Practical implications

The findings point to several practical implications for coach education, organizational development, and the sustainable provision of sport for athletes with VI.

- Coach education should include disability- and sport-specific modules that address non-visual communication, orientation, safety, sensory-responsive feedback, and motor learning for athletes with VI.
- Formal education should be complemented by supervised practical placements with experienced para-sport coaches. Such placements could create opportunities to connect theoretical knowledge with the context-specific and embodied knowledge required in everyday practice.
- Structured mentoring should be established for coaches entering para-sport. Mentoring may be especially important for accessing disability-specific knowledge, reflecting on coaching practice, building professional networks, and reducing the dependence of career entry on chance encounters (2, 3).
- Communities of practice and peer-learning opportunities should be supported across schools, clubs, federations, and Paralympic training centers. These structures could help document, share, and further develop experiential knowledge that currently remains largely embedded in individual biographies.
- Organizations should recognize the additional pedagogical and administrative work often undertaken by para-sport coaches, particularly by teacher-coaches who combine multiple professional roles. Adequate staffing, funding, and coordination structures are necessary if coaching expertise and sport development are to become sustainable rather than dependent on exceptional personal commitment.
- Collaboration among schools for students with VI, sports clubs, disability sport organizations, and specialized training centers should be strengthened. Such cooperation can support earlier recruitment, coordinated talent development (34), retention pathways, and access to specialist coaching and support personnel.

Taken together, these implications support an approach to professionalization that combines formal, informal, and experiential learning rather than privileging one mode over another. This is consistent with recent para-sport coaching research, which emphasizes the need for learning environments that integrate structured education, practical experience, and peer exchange (2–4).

### 4.5 Future research

Future research should examine how these three dimensions of professionalization—biographically contingent entry and learning, relational and impairment-specific expertise, and organizational support—interact across different sports, competition levels, and national or cultural contexts. Because the present findings are grounded in the German Paralympic sport system and in blind football, goalball, judo, and rowing, comparative work involving other disability groups, sport-specific contexts, and national systems would help clarify the transferability and limits of the identified patterns.

Further research should also investigate how coach education curricula can be adapted and evaluated in practice. This may include modular, evidence-based educational components addressing sensory-specific communication, adapted motor learning, practical safety arrangements, and organizational or administrative literacy. Longitudinal research could additionally explore how coaches' professional identities, knowledge, and networks develop across their careers, and whether formal mentoring, practice placements, or structured communities of practice reduce reliance on informal and contingent entry routes.

## 5 Limitations

This study has several limitations that should be acknowledged. First, the qualitative data set is limited in scope, both in terms of the number of interviews and the range of experiences captured, which constrains the breadth of phenomena that could be identified and limits the transferability of the findings beyond the specific contexts examined. Second, the sample covers only a small number of disciplines, so the results may not reflect coaching practices and structural conditions in other adaptive or Paralympic disciplines with different competitive histories and resource profiles. Third, the study was not designed as participatory research; coaches' perspectives are central, but the voices of athletes, parents, support staff and federation representatives are only indirectly present, which restricts the ability to triangulate interpretations and to incorporate stakeholder-led critique. Future research should complement these coach-based findings by including the perspectives of athletes with VI and other relevant stakeholders, such as parents, guides, support staff, and representatives of clubs and federations. Finally, all data were generated in relation to a single Paralympic training center and its associated networks, meaning that organizational patterns and role constellations described here may be specific to this site and its national context and cannot be assumed to be representative of other training centers or countries.

However, in light of the relatively small number of stakeholders and the limited visibility of coaching work in this domain, such an exploratory study provides an essential first step towards making these practices analytically accessible and offering an empirically grounded basis for future development in Paralympic sport for visually impaired athletes. This is why the limitations should be considered alongside several strengths. The study draws on information-rich accounts from experienced coaches with substantial domain-specific expertise across four Paralympic sports, enabling both cross-sport comparison and attention to sport-specific demands. The combination of a theoretically informed interview guide, cognitive pre-testing, reflexive field notes, carefully checked transcripts, and collaborative coding enhanced the transparency and interpretive robustness of the analysis. Moreover, the inductive–deductive analytic strategy enabled the study to relate the findings to established dimensions of coaching professionalization while remaining open to context-specific and unanticipated aspects of coaching athletes with vi. Finally, the focused institutional setting provided contextual depth and generated practically relevant insights into the interdependence of coaches' biographies, pedagogical practices, and organizational conditions.

## 6 Conclusion

Despite the substantial growth and increasing visibility of Paralympic sport (1), equal opportunities for participation and performance development remain far from assured for athletes with VI (35). Barriers related to accessibility, resources, specialist knowledge, and sustainable development pathways continue to shape their opportunities to enter, remain in, and progress within performance-oriented sport (7, 36). At the same time, research on the specific conditions of coaching and athlete development in this population remains limited (8). The present study therefore addresses an important gap at the intersection of inclusion, high-performance sport, and coach professionalization.

Rather than relying primarily on the commitment and experiential knowledge of individual coaches, organizations should create institutional arrangements that support the recruitment, mentoring, and retention of qualified coaches. The findings indicate that sustainable professionalization requires the integration of disability- and sport-specific education, mechanisms for transferring experiential knowledge, and reliable collaboration among schools, clubs, federations, and specialized support centers.

By identifying qualifications, pedagogical orientations, biographical pathways, structural conditions, and sport-specific adaptations relevant to coaching in performance-oriented sport for athletes with VI, this exploratory study contributes to answering the question of what professionalization in this field should entail. Given the small and context-specific sample, the findings do not constitute a universal model, but provide empirically grounded starting points for coach education, organizational development, and future research. Strengthening these conditions may not only improve coaching quality; it may also contribute to making high-performance sport more accessible, sustainable, and equitable for athletes with VI.

## Data Availability

The dataset presented in this article is not readily available because it consits of qualitative interview transcripts that contain personally identifiable information about the participants. Due to confidentiality agreements and ethical considerations, the dataset cannot be shared in order to protect the participants' privacy. Please direct any inquiries regarding access to the datasets to the 1st author. Please note that the dataset itself is not available due to confidentiality requirements. Requests to access the datasets should be directed to ulrich.theobald@ph-ludwigsburg.de.
